# Developing a surgical trial intervention protocol: using qualitative methods in the operating theatre

**DOI:** 10.1186/s13063-025-09088-y

**Published:** 2025-09-26

**Authors:** Maureen Twiddy, Richard Jackson, Kathryn Gordon, Julie Croft, Neil Corrigan, Deborah Stocken, Saba P. Balasubramanian

**Affiliations:** 1 Institute of Clinical and Applied Health Research, Hull York Medical School, University of Hull, Cottingham Road, Hull, HU6 7RX England; 2 Norfolk and Norwich University Hospital, Colney Lane, Norwich, NR4 7UY England; 3 Clinical Trials Research Unit, Leeds Institute of Clinical Trials, University of Leeds, Leeds, LS2 9JT England; 4 Sheffield Teaching Hospitals NHS Foundation Trust and University of Sheffield, Sheffield, S10 2RX England

**Keywords:** Randomised controlled trial, Qualitative research methods, Surgery, Intervention standardization, Consensus

## Abstract

**Introduction:**

Surgery is a complex intervention, so it is important to establish standards for both the standard and ‘novel’ procedures in randomized controlled trials (RCTs) that demonstrate that interventions are delivered as intended to fully understand and explain trial results. This study set out to identify and agree the key steps of a surgical intervention to be tested in the ‘near infrared fluorescent imaging in thyroid surgery’ (NIFTy) RCT to inform development of the surgical protocol, and trial materials.

**Method:**

Qualitative case studies of surgeries were undertaken prior to undertaking an RCT to evaluate the potential of a device to reduce post-surgical hypoparathyroidism. Each case study involved non-participant observation, video capture of total and completion thyroidectomies, and interviews with surgeons. A typology of operative steps was constructed. Two surveys were undertaken (1) to identify current practice around parathyroid identification; and (2) to determine surgeon views on the surgical steps. An international expert panel of six clinicians met to review findings and agree on the surgical steps (mandatory/optional) for operations in the RCT, including timing for use of fluorescence and the data items to be collected.

**Results:**

Ten case studies were undertaken. Video, observation and interview data found differences in surgical approach were driven only by pathology. A typology detailing the surgical steps and points where imaging could be used was developed. Sixty-four surgeons responded to survey 1; three-quarters always looked for parathyroid glands when operating. Forty surgeons responded to survey 2; capsular dissection of the thyroid lobe, preservation of parathyroid pedicle, and clinical assessment were important for parathyroid preservation. The expert panel agreed the key surgical components. These informed key data collection in NIFTy. Two specific surgical steps were strongly recommended and three mandated.

**Conclusion:**

Qualitative research in the operating theatre, prior to RCT allowed the identification of key components of the surgical intervention. The surveys and expert panel provided certainty about the acceptability of the surgical protocol and identified the core data to collect to evidence surgical decision making prior to embarking on the RCT. This qualitative process achieved clinical buy-in, improved trial conduct and allowed full explanation of the subsequent trial results.

**Trial registration:**

ISRCTN59074092. Registration date: 07/03/2022.

**Supplementary Information:**

The online version contains supplementary material available at 10.1186/s13063-025-09088-y.

## Introduction

Surgery is a complex healthcare intervention, meaning that there are multiple steps and components (e.g. interventions, techniques, surgical instruments). Surgeons can deliver these in different ways [4] and these can interact to affect outcomes. Surgeons may also adapt their surgical approach depending on the patient anatomy and pathology. This means that the ‘same’ surgical procedure can be undertaken in different ways. The Medical Research Council guidance on the development of complex interventions states that randomized controlled trials (RCT) should standardize the content and delivery of interventions [23]. When considering how much to standardize an intervention, such as thyroid surgery, it is important to balance being able to describe the intervention in sufficient detail for replicability, whilst not over-specifying the intervention to the extent that it reduces clinician acceptability in practice and of the trial results [14].

The SPIRIT statement [10] provides a checklist of items to be reported in trial protocols, and the TIDieR guidance [16] details aspects of the intervention to be reported, but neither of these checklists translate easily to the surgical setting. It is important to be able to describe the intervention in the clinical trial protocol to allow delivery and fidelity to be assessed. Blencowe and colleagues have developed an approach to enable clear description of surgical interventions; how it should be delivered, and the flexibility in surgical approach permitted [5]. They recommend that the key components/steps of the surgery are mapped, and decisions are made about whether, and to what extent, these need to be standardized, so they can be clearly documented in the trial protocol.

The present study adapts the qualitative case study approach developed by Blencowe and colleagues [4] to develop a surgical protocol and associated reporting mechanisms for use in the NIFTy trial. NIFTy is, a phase II/III RCT of a novel intervention (near infrared fluorescent (NIRF) imaging) during thyroid surgery to enable the research team to ensure the findings of the trial can be attributed to the use of autofluorescence and ICG (dye) fluorescence, rather than variation in surgical practice [11] (Trial registration ISRCTN59074092).

This qualitative study set out to determine which components and steps of thyroidectomy surgeons believe impact on the preservation of the parathyroid glands and which steps should be standardized or recorded in the trial. This would inform the design and delivery of the subsequent RCT to be able to demonstrate the interventions are delivered as intended, how they are delivered in real world practice and to be able to fully explain trial results. This paper describes the work undertaken to develop and agree a surgical protocol which ultimately achieved clinical buy-in and informed evidence generation that allowed mechanisms of effect to be examined, i.e. how does the device affect intraoperative decision making related to dissection of the thyroid and whether to auto transplant the parathyroid glands? [11].

## Methods

The preservation of the parathyroid glands during thyroid surgery may not only be affected by surgical experience and technique but also by specific approaches and interventions [2]. NIFTy is a UK phase II/III trial to determine the efficacy and effectiveness of near infra-red fluorescence (NIRF) imaging using autofluorescence and with ICG dye in reducing the risk of post-surgical hypoparathyroidism after bilateral thyroid surgery (NIHR 17/11/27). The use of NIRF may facilitate the identification and preservation of parathyroid glands during thyroid surgery, by providing real-time intra-operative visualization of parathyroid tissue [11]. In addition, the use of an exogenous dye allows for the assessment of parathyroid perfusion (and thereby viability) and for vessels feeding the glands to be identified. However, it is unclear how the technology will be used in practice, or whether the technology will change clinical decision-making. The primary outcomes are short (phase II) and long (phase III) term hypoparathyroidism damage after thyroid surgery.

**Fig. 1:**
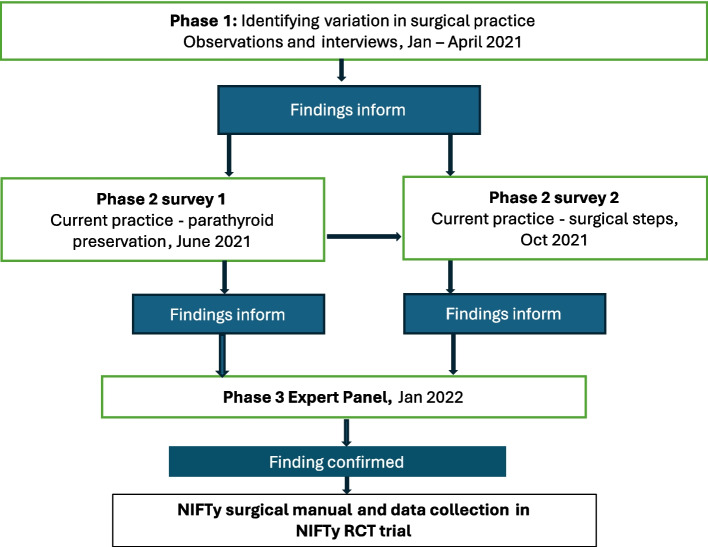
Qualitative flow diagram

The pre-RCT qualitative work described in this paper follows, builds on recommended methods [4] and comprises three phases: (1) mapping of the surgical steps of thyroidectomy in a single centre with a focus on parathyroid preservation; (2) surveys to understand wider practice in this area; (3) consensus methods to agree on the mandatory and optional surgical steps for the trial, data items to be recorded in the operative clinical report form (CRF), and the ways in which NIRF should and could be used within the trial (Fig. 1).

The study received ethical approval from East Midlands-Leicester Central Research Ethics Committee (20/EM/0062). Consent for video recording the surgical procedures was obtained from patients. Consent was obtained from all operating surgeons and theatre staff present at the time of the surgeries.

### Phase 1: Identifying the steps of a thyroidectomy and interventions to protect the parathyroid glands

To identify the steps of a total and completion thyroidectomy and examine how NIRF could be used in practice, a qualitative observational study was undertaken [3]. This consisted of non-participant observation and video recording of thyroidectomies, followed by semi-structured interviews with surgeons. The objectives were to (1) identify the steps of a total or completion thyroidectomy; (2) observe how NIRF was used in practice and (3) understand variation in practice that could affect parathyroid preservation.

#### Case study sampling strategy

We planned to recruit from three NHS Trusts and observe up to 10 surgeons. However, the COVID 19 pandemic limited our ability to recruit from two sites. We therefore undertook observations and video recordings of 10 surgeries undertaken by four surgeons at one NHS Trust (Table 1). Cases were selected to encompass varying degrees of difficulty. Data collection was stopped following analysis of these cases as saturation was reached and similarity in findings led us to conclude that further observations would be unlikely to yield more data.

**Table 1:** Parathyroid preservation interventions

Surgical intervention	(*N* = 38)*
Dissection of thyroid lobe—capsular dissection (ideally between the true and false capsule) at all times	Essential = 18 Very important = 18 Moderately important = 2 Little importance = 0 Not important = 0
Identification of the blood supply/pedicle to parathyroid	Essential = 10 Very important = 20 Moderately important = 7 Little importance = 1 Not important = 0
Clinical assessment of viability—inspection of colour alongside incision in doubtful cases and the use of techniques such as 100% oxygen and warm saline	Essential = 6 Very important = 13 Moderately important = 17 Little importance = 1 Not important = 1
Autotransplantation should be undertaken if the parathyroid glands are found to be ischemic at surgery	Essential = 8 Very important = 15 Moderately important = 11 Little importance = 4 Not important = 0

*38 respondents answered this part of the survey

#### Data collection

Non-participant observations of the surgeries were undertaken by RJ (medically qualified researcher) and MT (PhD qualified qualitative researcher) and documented using an observation schedule (appendix 1). Surgeons were asked to use the NIRF technology as they felt appropriate. Observations were supplemented by video data collected using a digital video camera on a tripod directed over the operating field; a head worn (go-pro) camera provided supplemental close-up images of the operating field. Recording started at skin incision and stopped at skin closure. The observation checklist was used to record real-time operative steps and activities within the operating theatre not picked up by the video. All recordings and notes were stored securely on the NHS Trust computer system.

Post operative semi-structured interviews with the primary surgeon were conducted by one or both researchers using a topic guide (appendix 2). Questions were open-ended to explore the surgeon’s thoughts about how the operation went, changes to the planned approach, and use of the NIRF device in practice. These were used to clarify the observational and video data. Interviews were audio-recorded and transcribed verbatim.

#### Data analysis

Data collection and analysis ran in parallel. Observation notes and interview recordings were transcribed. Each operation provided three sets of data: the digital video(s), operating theatre observations notes and surgeon interviews. Video analysis was undertaken by RJ and SB with each surgical step recorded on an Excel spreadsheet to give a clear account of the operation. The audio quality of the video was poor and so only visual data were used. The observational notes were mapped against the steps shown in the video to provide context.

An inductive descriptive approach was taken to the analysis of the interviews, enabling themes to be identified within the data [7, 12]. The interviews were imported into NVivo 11 (QSR International, Australia) to aid coding and analysis. Coding involved noting key phrases to capture the essence of the material. Coded material was grouped and re-grouped following subsequent interviews to identify themes. MT led the interview analysis with regular meetings with RJ and SB to discuss the coding and themes identified. As MT is a health services researcher and therefore an ‘outsider’ whereas RJ was a surgical trainee at that time of the data collection but not part of the thyroid team, each brought with them their own experiences to the analysis. Although a more experienced researcher, MT was new to thyroid surgery, so they understood the data at different levels, and so most interviews were undertaken by both researchers or by MT alone.

This approach enabled a comparison of the operating technique adopted by each surgeon and generated an understanding of the ‘usual’ steps, and variations in approach adopted. The interview findings were then mapped to the Excel data to relate these back to the video to explain and enhance understanding of variations in practice.

### Phase 2: Understanding clinical practice regarding parathyroid preservation during total or completion thyroidectomy

Two online surveys were developed. Survey 1 (appendix 3) asked about the number of thyroid operations performed each year. It used a binary response format to identify current practice for parathyroid gland identification and determining viability, and whether surgeons used NIRF technology.

Survey 2 (appendix 4) used the findings from the surgical observation study (phase 1) and survey 1 findings to ask about three areas: intra-operative interventions; surgical steps and importance of specific parathyroid preservation interventions. The survey used binary and 5-point Likert-style ordered categorical response formats to ask whether clinicians used the steps identified; and the importance of peri- and intra-operative steps and interventions used during a thyroidectomy. Surgeons were also asked what additional steps/interventions (if any) they utilise to preserve the parathyroid glands. Free text boxes were used to encourage clinicians to explain their answers. The survey incorporated clinical scenarios and videos demonstrating the use of autofluorescence and ICG dye to gain insight into the acceptability of the technology.

#### Data collection

Surveys were hosted by REDCap cloud a secure online system. Survey 1 was distributed to British Association of Endocrine and Thyroid Surgeons (BAETS) members in June 2021 via email. A reminder was sent two weeks later. Survey 2 was distributed in October 2021 using personal email invitations sent to respondents from survey 1 who consented to further involvement.

#### Data analysis

Descriptive statistics (median and range) were calculated for closed questions. Thematic content analysis [18] was conducted on the responses to the open-ended questions by one researcher (MT).

### Phase 3: Agreement of the surgical protocol and data to be collected in the operative clinical report form (CRF)

An expert panel with independent chair was convened to achieve consensus on the surgical protocol and data items to be collected in the operative CRF.

The expert panel comprised six individuals: an international expert on the use of NIRF, and experienced UK consultant surgeons from five NHS Trusts in the UK. Members were selected using a key informant approach [21] based on their clinical/research expertise. The research team attended to answer questions from the panel. A report of the observational study, and survey findings was circulated prior to the meeting. Members were asked to consider (a) the mandatory/optional aspects of the intervention; (b) steps taken to preserve the parathyroid glands; (c) the use of other surgical interventions (e.g. intraoperative recurrent laryngeal nerve (RLN) monitoring); (d) timing of autofluorescence and use of ICG during the trial and (e) level of detail to be provided in the intervention manual.

Members were asked to use the evidence to consider each surgical step and its relevance to the preservation of the parathyroid glands to determine any mandatory/optional steps and interventions to be used in the trial. The panel reviewed the evidence presented and discussed each step in turn until consensus was reached on the content of the operative CRF, including questions to assess clinical decision making before and after the use of AF and ICG, to determine if the device affected decision making. A written record of all decisions was made and used by the research team to write the surgical manual and operative CRF for use in the trial.

## Results

### What do clinicians do intra-operatively to identify and preserve the parathyroid gland?

Data from the observations, clinician interviews and surveys were used to answer this question.

All interviewees (phase 1) viewed it as important to locate the parathyroid glands as each lobe was mobilised, except for during cancer surgery, when the removal of cancerous tissue was prioritized. It was noted that even if parathyroid glands were viable at the mid-point in the surgery, this did not guarantee they would be viable at the end of the surgery, but interviewees believed that locating them provided the best possible opportunity to protect them. Surgeons were also asked if autofluorescence and ICG had affected clinical decision making, to inform the development of a form to record whether decision making changed following use of the device. The interviewees and survey respondents were supportive of the use of autofluorescence to aid parathyroid identification, to support clinical decision making, although this was as an adjunct to clinical experience.

*Interview 1: Well, on the right side we didn’t straight away see the parathyroid glands, either of them. But when the autofluorescence was on it pointed out two areas and we could see them. So, the autofluorescence was useful.*

*Interview 10.” I used the autofluorescence to make sure that the two glands were identified were definitely parathyroid glands, but I knew it anyway from the appearances of it.”*

Survey 1 (see additional file for data) investigated current parathyroid preservation practice. Sixty-three responses were received from across the UK and Northern Ireland (9% response rate). Views varied; 47/63 (75%) of respondents always looked for the parathyroid glands when performing thyroid surgery; the remainder usually/sometimes looked. Variations in practice were found as to *when* during surgery the parathyroid glands were looked for, the most common being: during upper and lower pole dissection only (19/63 :30%); 16/63 (25%) monitored closely, checking at initial mobilisation, upper/lower pole dissection and at end of surgery; 11/63 (17%) looked *only *at initial mobilisation.

Nearly two-thirds of respondents (41/63: 65%) reported that they assessed the viability of the parathyroid glands identified; 16/63 (25%) did this by appearance only; 8/63 (12%) did this by appearance, bleeding on incision and looking for feeding vessels; 17/63 (27%) did so by appearance and looking for feeding vessel. Only 26/63 (41%) reported they always evaluated the specimen after excision to see if there may be parathyroid glands attached to it; 9/64 (14%) never did this.

Survey 2 (see additional file for data) respondents were asked their views on the importance of a range of interventions used for parathyroid preservation (Table 1). Forty clinicians, from 34 NHS Trusts across the UK responded to at least part of the survey (64% response rate). All interventions were viewed as at least moderately important to almost all respondents, with capsular dissection viewed as the highest priority, followed by identification of the parathyroid blood supply.

### What are the surgical steps involved in a thyroidectomy?

The case study (phase 1) and survey 2 data were used to answer this question. Ten surgical cases were observed and contributed data (Table 2). Age and/or gender were not recorded for two cases. Patient ages ranged from 30 to 70 years; due to the small sample size actual age is not provided to avoid identification of individuals. Post operative interviews were conducted with three surgeons and lasted between 10 and 20 min. One operation was not videoed as the camera was not available; observational notes were made. Post operative interviews took place with clinicians following seven operations. Interviews could not be scheduled for two operations, and one recording failed.

**Table 2:** Summary of cases observed

Case	Operation/surgical team	Patient gender (M/F) and age group
1	Total thyroidectomy for large goitre O/S = consultant 1 A/S = associate specialist	F, 50–60
2	Completion right hemithyroidectomy with central neck dissection for primary thyroid cancer O/S = consultant 1 A/S = associate specialist	F, 30–40
3	Total thyroidectomy for Graves' disease O/S = consultant 2 A/S = associate specialist	M, age not recorded
4	Total thyroidectomy for large multi-nodular goitre and Graves' disease O/S = associate specialist A/S = consultant 1	F, 30–40
5	Completion right lobectomy + central neck dissection for papillary thyroid Ca pT3a O/S consultant 2 A/S = associate specialist	M, 70–80
6	Completion thyroidectomy with central neck dissection for thyroid cancer (details of cancer not provided) O/S = consultant 1 A/S = specialist trainee	M, 70–80
7	Total thyroidectomy for Graves’ disease O/S = Specialist trainee A/S = consultant 1	F, 20–30
8	Total thyroidectomy for Graves’ disease O/S = consultant1 A/S = consultant 2	missing
9	Total thyroidectomy for Graves’ disease O/S = specialist trainee A/S consultant 1 (swapped role during surgery)	F, 50–60
10	Completion thyroidectomy for thyroid cancer (details of cancer not provided) O/S = consultant 2 A/S = associate specialist (no video)	M, 70–80
*O/S* operating surgeon, *A/S* assisting surgeon		

The observations were used to map each surgical step to generate a clear description. Survey 2 was then used to find out if the surgical steps as described reflected wider clinical practice.

In the observational study (phase 1), each surgical step occurred in all cases, with only minor variations in practice stemming from differences in pathology and extent of surgery. Three variations were identified, each linked to a specific scenario (a) moving to dissection of the second lobe before the first lobe was entirely free to facilitate access; (b) larger incision when the thyroid extended into the chest cavity and (c) clearance of soft tissue in the central neck for a neck dissection. A crucial step identified by surgeons was mobilization of the lateral surface of the thyroid gland to visualize the parathyroid glands.

The surgical steps developed from the observational data are presented in Table 2, column 1. Column 2 presents the findings from Survey 2 which asked respondents which steps should be mandatory/optional in the trial and indicate if the step was unrelated to parathyroid preservation. Overall, respondents thought most steps should be mandatory, but variation in practice was found, suggesting that some flexibility was required. Free text comments were summarized and variation in practice was noted (column 3).

### Key step: use of ICG dye to identify parathyroid glands at end of surgery

Auto transplantation or parathyroid gland reimplantation involves transplanting fragments of a parathyroid gland that is no longer considered viable (due to loss of blood supply) into a muscle of the neck or the forearm to try to prevent long term hypoparathyroidism [19]. The first survey showed that only 27/63 (43%) of respondents would always auto transplant, 7/63 (11%) would never auto transplant.

### Expert panel consensus on standardization of thyroidectomy

The panel (phase 3) met online via MS Teams for one 2.5 hour meeting to review the findings and provide recommendations based on the evidence. All panel members were experienced thyroid surgeons, and most were experienced clinical trialists, but their knowledge and experience of NIRF in thyroid surgery varied, with one expert user, and two clinicians with some experience of the technology.

The survey respondents felt some steps should be mandated (Table 2 column 2). The expert panel discussed each surgical step in turn and concluded that mandating steps could reduce trial acceptability if it diverted from the surgeons’ usual practice. Consensus was reached through discussion and concluded that steps which provided an opportunity to preserve the parathyroid glands should be strongly recommended for both arms of the trial. One exception was the use of autofluorescence and ICG which is mandatory in the trial arm and prohibited in the control arm (see Table 2 column 4). Some surgical step descriptors were revised to improve clarity and reflect respondent feedback (shown in italics in Table 2). All interventions detailed in Table 3 were recommended practices for both arms of the trial and their use would be recorded.

**Table 3:** Main steps involved in a thyroidectomy

Operative steps (Phase 1 observational study data)	Survey 2 *N*=39	Variability in practice identified	Expert Panel Decision
Transverse or curvi-linear incision 6-8 cms in length approx. 2 finger breadths above clavicular heads; followed by elevation of sub-platysmal flaps; and division of strap muscles in midline.	Mandatory =13 Optional =16 Not relevant =10	No variability noted	Does not impact on parathyroid preservation. Not to be recorded
Dissect between the thyroid lobe and strap muscles as far as the carotid sheath. Separate the thyroid lobe from carotid sheath going down to pre-vertebral fascia.	Mandatory =26 Optional =9 Not relevant =4	No variability noted.	Strongly recommended as it provides better exposure and greater opportunity to preserve PG
Open carotid sheath to identify and confirm vagus nerve	Mandatory =1 Optional =25 Not relevant =13	Some surgeons do not do this routinely. Some do not use continuous nerve monitoring.	Not relevant – do not record
Step 1: At the upper pole control individual branches/tributaries separately at capsule. Step 2: Look out for external branch of superior laryngeal nerve and use nerve monitoring (if available) to confirm. *(Wording revised by expert panel for clarity)*	Mandatory =24 Optional =13 Not relevant =2	Not all respondents ligate branches individually. Mobilisation approach varies depending on the size & anatomy of the thyroid gland/goitre being removed. Not all routinely look for superior laryngeal nerve (SLN) May ligate arteries more proximal.	Step1 – mandatory. Record device used to control the individual branches. Step 2 -optional. Record how and if done.
Look out for the superior parathyroid behind upper pole	Mandatory =35 Optional =4	Not all do this, it depends on size of thyroid lobe.	Trial arm: mandatory to use AF and record if seen visually/with AF. Control arm: AF not to be used. Were PT looked for? Were they seen?
Aim to identify RLN, the inferior thyroid artery and parathyroids at this stage, if not identified before. Use nerve monitoring to help with RLN identification. *(Expert panel noted that not all use nerve monitoring.)*	Mandatory =23 Optional =15 Not relevant =1	Not all undertake recurrent laryngeal nerve (RLN) monitoring	Optional step. Was RLN identified? Was nerve monitoring used?
Continue with capsular dissection of the rest of the thyroid lobe. Ligate/divide feeding vessels ofinferior thyroid artery at entry into thyroid.	Mandatory =35 Optional 3 Not relevant =1	No variation noted	Strongly recommended unless central neck dissection undertaken.
Follow the above steps on the other side. Division of the thyroid gland (in midline) at this stage may be done to improve access. (*Expert panel agreed that all operations will include this unless central neck dissection, so no need to record)*	Mandatory =18 Optional =16 Not relevant =5		No need to record
Examine the specimen to look for inadvertent parathyroidectomy. If presence of parathyroids is suspected, consider excision and auto-transplantation	Mandatory =33 Optional =5 Not relevant =1		Essential to check for inadvertent parathyroidectomy For intervention arm: was AF used? Was PT seen on specimen?
Start ipsilateral dissection on side of tumour (if unifocal). Remove all fatty tissue, lymph nodes and fascia taking care to avoid injury to the RLN, any identified parathyroids along with their blood supply. Use AF (in the trial arm) as required. Complete contralateral dissection (opposite side of tumour) if appropriate using AF (in the trial arm) as required. *Expert panel noted optional step when central neck dissection is indicated). *	Mandatory = 27 Optional =12	Not routinely undertaken unless recommended at MDT.	Optional step if central neck dissection indicated. Trial arm: Use AF to confirm presence of inferior PT. Use to check is PT on pedicle.
Secure haemostasis in the standard manner. Look at previously identified parathyroids in the thyroid bed and in case of ischaemic looking or non-viable parathyroids, consider auto-transplantation. Use ICG fluorescence (in the trial arm) to assess viability. *Expert panel agreed scoring approach for CRF – yes, unsure, no)*	Mandatory = 33 Optional = 6	Should assess viability – but if in situ, some leave and do not routinely autotransplant. If inadvertently removed, some autotransplant. In favour of leaving in situ and assessing using AF.	Trial arm: ICG to be mandated. Score each PT and pedicle. Has ICG changed clinical decision? Control arm: usual practice Both arms – check each PT (colour/profusion).

The information gathered directly informed the development of the NIFTY surgical manual and operative CRF to allow accurate monitoring of how closely surgeons followed the surgical guidance and complied with the trial protocol, including evidencing when, and if, the use of autofluorescence and ICG changed clinical decision making.

## Discussion

This study used qualitative case studies and survey data to identify the key steps of a thyroidectomy to inform the surgical protocol for the NIFTy RCT. The case studies used operating theatre observations, video data and interviews with surgeons to explore variation in clinical practice. These initial findings were then tested out using survey methods and an expert panel.

The study provided important insights into how the procedure can vary between patients but also found that relatively few of the key steps of a thyroidectomy were thought to directly impact on parathyroid preservation. There was general support from the clinical community for restricting practice in the trial, but the expert panel members were concerned about this reducing trial recruitment, so strongly recommended practices but limited the mandatory steps to those directly related to using the device. Instead, all data would be recorded in the operative CRF so that it would be possible to capture any variance with how operations are carried out in the RCT.

There was significant variation in practice around parathyroid identification during surgery, and steps taken for their preservation. Only three quarters of surgeons reported that they always looked for the parathyroid glands during surgery, despite the risk of post-surgical hypoparathyroidism after bilateral thyroid surgery [9, 13, 20] and respondents varied in the time-points within the procedure that they looked for the glands. Hypoparathyroidism has a significant impact on patients’ quality of life [8] and the potential for kidney damage [15], accumulation of calcium in the tissues [25] and seizures is significant [24] and can be life threatening [1]. Although clinicians who responded to the survey were supportive of the use of autofluorescence and ICG dye to aid parathyroid identification, they were viewed as an adjunct to clinical experience.

Well-designed and conducted RCTs provide strong evidence to determine the efficacy and effectiveness of interventions. The Consolidated Standards of Reporting Trials (CONSORT) statement [22] provides a checklist for reporting RCTs, with a specific extension designed for nonpharmacologic trials (CONSORT-NPT) [6]. The TIDieR framework [16] provides guidance for reporting intervention duration, dose and materials used. However, guidance specific to surgical interventions does not exist. Clear reporting of the surgical interventions is necessary to determine whether the findings of the trial can be attributed to the intervention, components of the intervention or variation in surgical practice. The clinician surveys and expert panel concluded that many of the surgical steps involved in a total or completion thyroidectomy were unrelated to the preservation of the parathyroid glands. This opened the opportunity for a compromise position and the expert panel therefore opted to balance the need for adequate standardization with practicality and record the use of all interventions which were thought to be linked to parathyroid preservation. This resulted in the development of a surgical protocol that reflects real-world clinical practice and the production of a detailed operative CRF to inform data collection of key clinical steps, rather than tight mandating of the surgical steps.

Drawing on, and adapting the approach developed by Blencowe and colleagues [4] allowed us to develop a surgical protocol that identified the key surgical steps and the interventions linked to parathyroid preservation that were agreed upon and could be delivered in trial centres. The benefits of the qualitative case study methodology were that the steps of the intervention could be clearly mapped using empirical data. The video recordings supplemented by interviews enabled the researchers to gain a clear insight into how the intervention could be used in clinical practice [3].

The response rate to survey 1 was relatively low and reflective of those who were interested in this topic, and so there may be viewpoints not reflected in our data, and so there may be some bias in our data. We found only modest variation in the operative steps, and the survey data largely reflected the observation data, except for some variation in a few surgical steps. In contrast, surgeons varied significantly in their reported intra-operative management of the parathyroid glands, which was the focus of this study. This suggests that although the number of survey responses received was low, the survey largely achieved its goal which was to identify the variation in practice that needed to be captured by the CRF in the operating theatre. We do acknowledge that there may be other approaches or combinations of actions which were not captured in the data.

COVID severely restricted the number of sites and surgeons we could observe, and the video technology available to us provided poor audio quality, so much of the detail came from the observational notes and interviews, and some nuance may have been lost. The inclusion of a single site and four surgeons allowed us to map some variation in surgical procedure and allowed us to collect naturalistic empirical data [4, 17]. However, our inability to observe first-hand the variation in surgical practice between NHS Trusts and across thyroid and ENT surgeons is a limitation to our study. We tried to mitigate the effect of the lack of observational data by the inclusion of the surveys which allowed us to gain wider consensus and demonstrated that the surgical steps identified by the qualitative work resonated with the clinical community. Using data from the surveys and expert panel meant that the boundaries of the surgical protocol were informed by the wider surgical community, and the study identified the data needed to be collected, to identify the impact autofluorescence and ICG had on clinical decision making. The results of the survey and expert panel decision will be validated in the clinical trial when we will examine whether the anticipated range of surgical actions to protect the parathyroid glands intra-operatively are replicated in practice.

## Conclusion

Surgery specific protocols are rarely developed in randomised clinical trials of surgical interventions. This qualitative study extended an approach pioneered by Blencowe et al. [5]. The case study methodology allowed insights into the procedures and interventions that affect parathyroid preservation and informed the development of the surgical protocol and data collection for the trial. We found similarities in how surgeons conduct the procedure, but significant differences in the interventions and steps adopted to preserve the parathyroid glands.

The findings provided insight into when and how autofluorescence and ICG fluorescence would be used in the trial, and these findings informed the trial specific surgical protocol and data items on the operative CRF. The decision of the expert panel is not to mandate the surgical steps but to record these. The trial will examine the range of approaches used to protect and preserve the parathyroid glands during surgery. The findings will elucidate what impact these actions and the use of ICG have on parathyroid preservation.

This methodology provides a mechanism to define and describe complex surgical interventions, develop an acceptable trial specific surgical protocol, evidence how they are delivered in practice to improve trial conduct and fully understand and explain trial results.

## Supplementary Information

Supplementary Material 1.

Supplementary Material 2.

Supplementary Material 3.

Supplementary Material 4.

## Data Availability

The anonymized survey datasets generated and analysed during the current study are available as a supplementary file. Interview transcripts are not available due to the small sample and single site design.
