# Supplementary material for: Developing a surgical trial intervention protocol: using qualitative methods in the operating theatre
Source: Trials. 2025 Sep 26;26:368. doi: 10.1186/s13063-025-09088-y (PMC12465219; doi:10.1186/s13063-025-09088-y)
Supplement: Supplementary file 1 — Supplementary Material 1. [file 13063_2025_9088_MOESM1_ESM.docx]

**Observation schedule - NIFTy**

**Time patient arrived: KTS: Finish time:**

**People in room: Patient factors:**

**Planned operation & indication:**

| Time/Phase of operation | Surgeons | Anaesthetists | Nurses |
| --- | --- | --- | --- |
| Patient positioning/anaesthetic   - Number of cannulae/Separate cannulae for ICG/ changes or additional cannulae - Patient & neck position (shoulder roll/ head ring /supports etc). Positional adjustments performed. - Pre-op or post-operative nerve block (type, method, Infiltration used, amount, uni/bilateral) - Intra-operative nerve monitoring (Yes/No, type, Continuous/Intermittent)   TECHNICAL  - Communication  - Progress of stage  - Economy of movement  - Instruments/tissue handling  CONTEXT  - Operating surgeon at this step (consultant/SpR/other)  - Surgeons position during stage  - Patient factors  - Surgeon factors;  (Tiredness/ stress)  - Equipment  - General Environment;  (Distractions/interruptions  /music/noise) |  | | |

**Observation schedule**

| Time/Phase of operation | Surgeons | Anaesthetists | Nurses |
| --- | --- | --- | --- |
| Opening of neck   - Skin incision site, length, position, anatomical landmarks used for incision - Method for skin incision and dissection through deeper tissues (knife/harmonic/   monopolar/bipolar)   - Extent of subplatysmal flaps (which level used) - Haemostasis management, anterior jugular veins encountered +/-ligated   TECHNICAL  - Communication  - Progress of stage  - Economy of movement  - Instruments/tissue handling  CONTEXT  - Operating surgeon at this step (consultant/SpR/other)  - Surgeons position during stage  - Patient factors  - Surgeon factors;  (Tiredness/ stress)  - Equipment  - General Environment;  (Distractions/interruptions  /music/noise) |  | | |

**Observation schedule**

| Time/Phase of operation | Surgeons | Anaesthetists | Nurses |
| --- | --- | --- | --- |
| Mobilisation of thyroid lobe – first side   - Extent of anterolateral dissection (carotid sheath/pre-vertebral fascia/Middle Thyroid vein) - Techniques/instruments used (Scissors/bipolar/   Harmonic)   - Sequence of steps - Systematic (Yes/No) - When were parathyroids seen or looked for, if so which? (Upper – when mobolising upper pole, Lower- when identifying RLN & artery) - When was RLN seen or looked for? - When was AF used and what were findings? - When was DF used, dose, number of times and what were findings?   TECHNICAL  - Communication  - Progress of stage  - Economy of movement  - Instruments/tissue handling  CONTEXT  - Operating surgeon at this step (consultant/SpR/other)  - Surgeons position during stage  - Patient factors  - Surgeon factors;  (Tiredness/ stress)  - Equipment  - General Environment;  (Distractions/interruptions  /music/noise) |  | | |

**Observation schedule**

| Time/Phase of operation | Surgeons | Anaesthetists | Nurses |
| --- | --- | --- | --- |
| Mobilisation of thyroid lobe – second side   - Extent of anterolateral dissection (carotid sheath/pre-vertebral fascia/Middle Thyroid vein) - Side surgeon stands on - Techniques/instruments used (Scissors/bipolar/   Harmonic)   - Systematic (Yes/No) - Sequence of steps - When were parathyroids seen or looked for? If so which? (Upper – when mobolising upper pole, Lower- when identify RLN & artery) - Which ones were seen? - When was RLN seen or looked for? - When was AF used and what were findings? - When was DF used, dose, number of times and what were findings?   TECHNICAL  - Communication  - Progress of stage  - Economy of movement  - Instruments/tissue handling  CONTEXT  - Operating surgeon at this step (consultant/SpR/other)  - Surgeons position during stage  - Patient factors  - Surgeon factors;  (Tiredness/ stress)  - Equipment  - General Environment;  (Distractions/interruptions  /music/noise) |  | | |

**Observation schedule**

| Time/Phase of operation | Surgeons | Anaesthetists | Nurses |
| --- | --- | --- | --- |
| *Central neck dissection – first side*   - Approach to dissection - Extent of dissection (levels 6 +/- 7), lateral limits (medial or lateral to RLN), Pre-tracheal/para-tracheal   Nodes removed   - How were parathyroids managed during dissection (dissection onto a pedicle/   Auto-transplantation/ unclear (enbloc resection)   - When was AF used, number of times and what were findings? - When was DF used, dose, number of times and what were findings?   TECHNICAL  - Communication  - Progress of stage  - Economy of movement  - Instruments/tissue handling  CONTEXT  - Operating surgeon at this step (consultant/SpR/other)  - Surgeons position during stage  - Patient factors  - Surgeon factors;  (Tiredness/ stress)  - Equipment  - General Environment;  (Distractions/interruptions  /music/noise) |  | | |

**Observation schedule**

**People in room:**

**Music:**

| Time/Phase of operation | Surgeons | Anaesthetists | Nurses |
| --- | --- | --- | --- |
| *Central neck dissection – second side*   - Approach to dissection - Extent of dissection (levels 6 +/- 7), lateral limits (medial or lateral to RLN), Pre-tracheal/para-tracheal   Nodes removed   - How were parathyroids managed during dissection (dissection onto a pedicle/   Auto-transplantation/ unclear (enbloc resection)   - When was AF used, number of times and what were findings? - When was DF used, dose, number of times and what were findings?   TECHNICAL  - Communication  - Progress of stage  - Economy of movement  - Instruments/tissue handling  CONTEXT  - Operating surgeon at this step (consultant/SpR/other)  - Surgeons position during stage  - Patient factors  - Surgeon factors;  (Tiredness/ stress)  - Equipment  - General Environment;  (Distractions/interruptions  /music/noise) |  | | |

**Observation schedule**

| Time/Phase of operation | Surgeons | Anaesthetists | Nurses |
| --- | --- | --- | --- |
| Closure   - LA infiltrated or Nerve block (type/amount/location) - Methods for haemostasis - Use of valsalva - Method of closure – layers, suture type - Use of drain (type) - Skin dressing   TECHNICAL  - Communication  - Progress of stage  - Economy of movement  - Instruments/tissue handling  CONTEXT  - Operating surgeon at this step (consultant/SpR/other)  - Surgeons position during stage  - Patient factors  - Surgeon factors;  (Tiredness/ stress)  - Equipment  - General Environment;  (Distractions/interruptions  /music/noise) |  | | |
