# Supplementary material for: Developing a surgical trial intervention protocol: using qualitative methods in the operating theatre
Source: Trials. 2025 Sep 26;26:368. doi: 10.1186/s13063-025-09088-y (PMC12465219; doi:10.1186/s13063-025-09088-y)
Supplement: Supplementary file 2 — Supplementary Material 2. [file 13063_2025_9088_MOESM2_ESM.docx]

**NIFTy Topic guide (post surgery)**

Repeat introduction and thank the surgeon for participating, reinforce confidentiality.

Ask for permission to audio record interview.

As discussed, we are trying to understand the complexity of surgical interventions within trials. I would like to ask you just a couple of questions about how this [ patient X] surgery went.

**Questions**

1. Can you talk me through how the surgery went? Was this an easy/difficult surgery? Why? prompt: e.g retrosternal extension, central neck dissection, size of thyroid gland, presence of Grave’s disease
2. Were there any planned modifications to your usual approach for this patient? If yes, what were they and why?
3. Were there any unusual or unexpected steps with patient X?
   1. If yes, what were they and what did you do, and why?
4. You decided to use NIRF at [point(s) X, Y Z ], can you tell me why you decided to use it then?
   1. What guided that decision? surgical expertise, size/ location of glands, pathology
5. What information did you get from the imaging?
   1. What impact, if any do you think this had on your surgical decision making?
   2. Were you able to save the para-thyroid glands?

END

Ask for sociodemographic details, grade and experience (i.e number of previous cases performed). Thank the surgeon again and re-iterate confidentiality
