# Supplementary material for: Developing a surgical trial intervention protocol: using qualitative methods in the operating theatre
Source: Trials. 2025 Sep 26;26:368. doi: 10.1186/s13063-025-09088-y (PMC12465219; doi:10.1186/s13063-025-09088-y)
Supplement: Supplementary file 4 — Supplementary Material 4. [file 13063_2025_9088_MOESM4_ESM.docx]

**Survey of surgeons on specific interventions and steps during thyroid surgery and their relevance to parathyroid preservation**

The NIFTy Trial (Near Infrared Fluorescence (NIRF) Imaging to prevent Post-surgical Hypoparathyroidism (PoSH) after Thyroid Surgery) will open to recruitment shortly. This is a phase II/III pragmatic, multicentre randomised controlled trial and compares NIFR imaging with ICG against usual practice, in reducing the risk of post-surgical hypoparathyroidism after total or completion thyroid surgery.

Before the trial opens we need to better understand ‘usual practice’ and understand which interventions are commonly used, and of these which should be permitted within the trial. We also need to understand which inter-operative steps are believed to influence the development of PoSH.

This survey will take you through the key surgical steps for a ‘standard’ thyroidectomy (removal of first pole only and central neck dissection when indicated). You will be asked whether you do this step, if you feel that the step contributes to a successful outcome, and whether for the trial this step should be mandatory or optional.

A key feature of NIFR is the ability to harness the parathyroid glands ability to auto fluoresce, which enables better visualisation of the PT in the surgical field. There are several points in the surgery where this technique could be used. As we take you through the surgical steps we will ask you whether you think this approach would be useful at this point.

[insert image to show Autofluorescence] *Caption: This is an image of the parathyroid glands captured using autofluorescence.*

We will then show you some still images of a couple of surgeries where we used autofluorescence (AF) and will demonstrate the use of ICG dye to illuminate the parathyroid glands.

The survey will take approximately 25 minutes to complete.

**The results will be used to help us to develop the surgical protocol to be used in the trial.**

Any questions please contact Dr Saba Balasubramanian ([sabapathy.balasubramanian@nhs.net](mailto:sabapathy.balasubramanian@nhs.net)) or Dr Maureen Twiddy (maureen.twiddy@hyms.ac.uk)

**The role of specific interventions before, during and after thyroid surgery**

**Below is a list of intra-operative interventions. Do you use these in your practice now? In a trial should we control whether or not surgeons use any of these interventions?**

| **Specific intervention** | **Always / often / sometimes / rarely / never** | **Please comment on when you would do this if you have indicated ‘often’, ‘sometimes’ or ‘rarely’** | **In a trial evaluating fluorescent imaging for parathyroid preservation in thyroid surgery, would you consider this intervention mandatory, optional or ‘not-relevant’?** |
| --- | --- | --- | --- |
| Intravenous steroids at induction |  |  |  |
| Intraoperative RLN monitoring |  |  |  |
| Local anaesthetic infiltration at the incision site before or after surgery |  |  |  |
| Superficial cervical plexus block before or after surgery |  |  |  |
| Use of tranexamic acid |  |  |  |
| Use of loupes to magnify the surgical field |  |  |  |
| Routine identification of the external laryngeal nerve |  |  |  |
| Use of drains |  |  |  |
| Routine autotransplantation |  |  |  |

**Mandatory** = You think this intervention contributes to the preservation of the parathyroid glands. All patients randomised to the trial (intervention or control arm) should receive this, regardless of presentation**. Optional** = It is not clear whether this intervention contributes to the preservation of parathyroid glands and surgeons so should be able to choose whether or not to do this, depending on presentation. Surgeons will be asked to record this on the surgical checklist. **Not relevant** = you are sure this intervention does not contribute to the preservation of the parathyroid glands and so does not need to be recorded for the trial.

**Operative Procedure (pertaining to first time, traditional trans-cervical open surgery)**

**In the next section we will run through the surgical steps, pertaining to a first time, traditional trans-cervical open surgery. We want to know if your approach differs, and whether in the trial you would be happy to follow the approach suggested or whether there should be flexibility. Keep in mind that the more flexibility, the more that will need to be recorded on a CRF.**

| **Specific steps of surgery** | **Suggested approach** | **Please detail if your approach deviates from what is suggested in any significant way (open text box)** | **Any additional comments on this step with a focus on parathyroid identification and/or preservation** | **In a trial evaluating fluorescent imaging for parathyroid preservation in thyroid surgery, would you consider the suggested approach *to be mandatory, optional or ‘not-relevant’?** |
| --- | --- | --- | --- | --- |
| Neck opening | Transverse or curvi-linear incision 6-8 cms in length approx. 2 finger breadths above clavicular heads; followed by elevation of sub-platysmal flaps; and division of strap muscles in midline |  |  |  |
| Exposure of thyroid lobe | Dissect between the thyroid lobe and strap muscles as far as the carotid sheath.  Separate the thyroid lobe from carotid sheath going down to pre-vertebral fascia. |  |  |  |
| Exposure of thyroid lobe | Look out for any parathyroid glands that are obviously visible. |  |  |  |
| Exposure of vagus | Open carotid sheath to identify and confirm vagus nerve |  |  |  |
| Mobilisation of upper pole | Start with upper pole and ligate individual branches/tributaries separately at capsule; Look out for external branch of superior laryngeal nerve and use nerve monitoring (if available) to confirm |  |  |  |
| Mobilisation of upper pole | look out for the superior parathyroid behind upper pole. (with or without autofluorescence) |  |  |  |
| Exposure and identification of the RLN and the parathyroid glands | Aim to identify RLN, the inferior thyroid artery and parathyroids at this stage, if not identified before.  Use nerve monitoring to help with RLN identification |  |  |  |
| Exposure and identification of the RLN and the parathyroid glands | Use autofluorescence (if available) to help in parathyroid identification |  |  |  |
| Continue capsular dissection | Continue with capsular dissection of the rest of the thyroid lobe.  Ligate/divide feeding vessels of inferior thyroid artery at entry into thyroid |  |  |  |
| Continue capsular dissection | Use autofluorescence (if available) to help in parathyroid identification |  |  |  |
| Continue with lobectomy on the other side | Follow the above steps on the other side.  Division of the thyroid gland (in midline) at this stage may be done to improve access. |  |  |  |
| Specimen removal | Check for AF on the specimen to look for inadvertent parathyroidectomy.  If presence of parathyroids are suggested on AF, consider excision and auto-transplantation |  |  |  |
| Central neck dissection | Start ipsilateral dissection on side of tumour (if unifocal).  Remove all fatty tissue, lymph nodes and fascia taking care to avoid injury to the RLN, any identified parathyroids along with their blood supply. Complete contralateral dissection (opposite side of tumour) if appropriate using AF (if available) as required. |  |  |  |
| Central neck dissection | Use AF (if available) as required during the neck dissection. |  |  |  |
| Evaluation of bed | Secure haemostasis in the standard manner.  Look at previously identified parathyroids in the thyroid bed and in case of ischaemic looking or non-viable parathyroids, consider auto-transplantation.  Use ICG fluorescence (if available) to assess viability |  |  |  |
| Wound closure | Use standard closure methods |  |  |  |

*Mandatory means surgeons would have to follow the approach suggested, including all steps. Optional means surgeons could choose if/how they do this, but would be asked to record this in the operation log. Not relevant means surgeons would not be asked to record if this step is performed or not as it is not relevant to outcomes.

PRESERVATION OF PARATHYROIDs

| Parathyroid preservation can be carried out in different ways. Some of the following relate to usual practice, and some questions are specific to the use of AF. Please tell us whether in a trial you think these steps should be mandatory or not. **Method / intervention** | **Suggested approach and rationale** | **Comments** | **In a trial evaluating fluorescent imaging for parathyroid preservation in thyroid surgery, would you consider this intervention mandatory, optional or ‘not-relevant’?** |
| --- | --- | --- | --- |
| Colour of drapes | We suggest using Green drapes (to help with contrast in fluorescence |  |  |
| Dissection of thyroid lobe | Capsular dissection (ideally between the true and false capsule) at all times |  |  |
| Identification of the blood supply/pedicle to parathyroids | This is not required but identification of a pedicle can help in avoiding devascularisation |  |  |
| Early use of ICG fluorescence prior to or during dissection of thyroid lobe to map the vascular pedicle of the parathyroid | This may hinder later use of auto-fluorescence. Given this, would you want the option of using ICG early in the surgery? |  |  |
| Clinical assessment of viability | Inspection of colour alongside incision in doubtful cases  Use of 100% oxygen, warm saline etc. |  |  |
| Autotransplantation | In cases of ischaemic glands, remove the gland and place in cold saline. At end of procedure, mince the gland, inject/place the sample into a pocket of vascularised skeletal muscle. You may draw into a 1 ml syringe and inject into muscle. |  |  |

**Further questions (videos were provided to respondents as stimulus material)**

1. Show naked eye and **AF identification of the** **superior parathyroid gland** (video/still images). Explain the context.
   1. Do you think that AF is a useful tool at this stage to aid the identification of the superior gland? Definitely / maybe / not sure / unlikely / definitely not
   2. Please use the text box to comment _________________________________
2. Show naked eye and **AF identification of the inferior parathyroid gland** (video/still images). Explain the context.
   1. Do you think that AF is a useful tool at this stage to aid the identification of the superior gland? Definitely / maybe / not sure / unlikely / definitely not
   2. Please use the text box to comment _________________________________
3. Show vascularisation of a parathyroid gland with **ICG fluorescence** (colour video/still images, AF and ICGF). Explain the context. Use an example where the **parathyroid gland is viable**.
   1. Do you think ICG fluorescence is a useful tool at this stage to assess the viability of the parathyroid gland? Definitely / maybe / not sure / unlikely / definitely not
   2. Please use the text box to comment _________________________________
4. Show vascularisation of another parathyroid gland **with ICG fluorescence** (colour video/still images, AF and ICGF). Explain the context. Use an example where the **parathyroid gland is viable.**
   1. Do you think ICG fluorescence is a useful tool at this stage to assess the viability of the parathyroid gland? Definitely / maybe / not sure / unlikely / definitely not
   2. Please use the text box to comment _________________________________
5. Show vascularisation of another **parathyroid gland with ICG fluorescence** (colour video/still images, AF and ICGF). Explain the context. Use an example where **the parathyroid gland is not viable** on fluorescence angiogram but looks fine on naked eye inspection**.**
   1. Do you think ICG fluorescence is a useful tool at this stage to assess the viability of the parathyroid gland? Definitely / maybe / not sure / unlikely / definitely not
   2. Please use the text box to comment _________________________________
   3. Would you auto-transplant this gland that does not look viable on ICG fluorescence imaging? Definitely / maybe / not sure / unlikely / definitely not
   4. Please use the text box to comment _________________________________
6. In your opinion, what are the important surgical steps that contribute to parathyroid preservation during thyroid surgery?

Open text box

1. In your opinion, what steps or instruments (such as diathermy) are to be avoided or used with care so as to preserve the parathyroid glands during thyroid surgery?

Open text box

1. How do you feel the use of auto-fluorescence and ICG fluorescence would affect the way you perform thyroid surgery?

Open text box
